# Effect of Microwave-Assisted Curing on Properties of Waterborne Silicone Antifouling Coatings

**DOI:** 10.3390/polym14214493

**Published:** 2022-10-24

**Authors:** Meng Li, Zhanping Zhang, Yuhong Qi

**Affiliations:** Department of Materials Science and Engineering, Dalian Maritime University, Dalian 116026, China

**Keywords:** microwave, silicone, ship, waterborne coatings, antifouling coating

## Abstract

Waterborne silicone coatings are prepared in this paper by using silicone emulsion as a film-forming material, γ-methacryloxypropyltrimethoxysilane, and dibutyltin dilaurate as a curing agent and a catalyst, respectively. The corresponding coatings are obtained by controlling different microwave times to accelerate the coating curing. The surface morphology, roughness, surface properties, mechanical properties, and antifouling properties of the coating are studied by laser confocal microscope, contact angle measurement, tensile test, marine bacterial attachment test, and benthic diatom adhesion test. Additionally, the action mechanism of microwaves in the curing process of the coatings is also discussed. The results show that the microwave can greatly reduce the curing time of waterborne silicone coating. It can improve the painting efficiency, the surface roughness of the coating, and the mechanical properties of the coatings. The change in roughness increases the contact angle of the coating, reduces the apparent surface energy, and then improves the antifouling performance. For the coating cured by microwave, with the increase in microwave curing time, the water and diiodomethane contact angles of the coating gradually increase, and the surface energy gradually decreases from about 20 mJ/m^2^ to 10.8 mJ/m^2^. With the increase in microwave time, the attachment amount of *Navicular Tenera* gradually decreases, the removal rate gradually increases, and the removal rate of *Navicular Tenera* in the coating increases from 15.36% to 31.78%. The bacterial removal rate of the coating can be increases from 11.05% to 22.28% after microwave curing. Microwave-assisted curing is helpful in improving the antifouling and self-cleaning performance of waterborne silicone coatings, showing promising potential applications.

## 1. Introduction

There are many fouling organisms in the ocean. These organisms will adhere to the surface of the ship, increasing the drag and fuel consumption of the ship. They will also adhere to the surface of other marine equipment, which will affect the life and safety of the equipment, resulting in major economic problems [1,2]. With the development of antifouling coatings, these problems have been gradually solved. However, the traditional metal-based antifouling coatings have been prohibited or limited because the coatings are toxic, which pollutes the seawater and affects the marine ecology [3,4,5]. Therefore, environmentally friendly antifouling coatings have become an important research direction [6,7].

Silicone rubber has low viscosity, ease of processing, high heat and acid aging resistance, and good flexibility [8]. The strong chemical bonding inherent within their siloxane networks as well as their thermal stability promote inertness against chemical attack. Therefore, silicone rubber has the potential for anticorrosion, anti-biofouling, anti-icing, flame-resistance, self-cleaning, as well as anti-reflection [9]. The coating made from PDMS has been proven to be a promising environmentally friendly antifouling coating [2]. The fouling organisms are not firmly attached to it and can be easily removed under the scouring water flow, which makes silicone coatings have good antifouling performance [7].

At present, silicone antifouling coatings have been widely studied. For example, Mohamed et al. [10] prepared a polydimethylsiloxane elastic nanocomposite with graphene oxide sheets and TiO_2_ nanorods on its surface. Compared with silicone coating, this coating has a rough topology and low surface free energy, showing super-hydrophobicity and excellent antifouling performance while the mechanical properties of the coating are not reduced. Mohamed et al. [11] prepared an elastic silicone/GO-Al_2_O_3_ hybrid nanorods sheet coating. Well-dispersed GO-γ-Al_2_O_3_ nanorod sheet mixture increases the contact angle of the coating, reduces the surface energy, and provides micro-nano-roughness. Due to the above changes, the coating obtained super-hydrophobicity and high thermal stability. Xue et al. [12] prepared a self-layered coating with acrylic resin and silicone. Compared with the original silicone coating, this coating still has the same antifouling performance, but the adhesive strength has improved. Additionally, experiments showed that the mechanical properties of this coating are enhanced and very stable in different environments. This research provides a simple and easy self-stratification strategy for developing antifouling coatings.

Traditional silicone coatings are solvent-based coatings; that is, coatings contain volatile organic compounds (VOCs), which are harmful and pose a threat to the ecological environment and human health [13]. Waterborne coatings can eliminate the impact of VOCs on the environment. Therefore, waterborne silicone coatings will become an important research direction. So far, waterborne silicone coatings have been studied in various industries. For example, Wang et al. [14] used acrylic elastic emulsion, pure silicone resin, and polyurethane dispersion as the base material, rutile titanium dioxide, other materials as pigments and fillers, and some auxiliaries to prepare waterborne silicone coatings. Tests show that the coating has high strength, good antifouling, and breathability. Zhu et al. [15] mixed black infrared reflective pigment, a small amount of SiO_2_ nanoparticles, and fluorinated acrylic emulsion in waterborne silicone emulsion, and the mixture was applied to an aluminum plate to prepare the coating. Such a coating has the characteristics of high infrared reflection and super-hydrophobicity. Because of its super-hydrophobicity, the coating has a good self-cleaning ability and can still maintain this performance for a long time. Liu et al. [16] studied the effects of a cationic emulsifier (Octadecyl trimethyl ammonium bromide, OTAB) and an anionic emulsifier (Sodium dodecyl benzene sulfonate, SDBS) on the properties of the waterborne silicone coating. The test results showed that although the surface free energy of the coatings is hardly affected by the type of emulsifier, the waterborne silicone coatings prepared by cationic silicone emulsion have higher crosslinking density, a smoother surface, and better comprehensive mechanical properties and antifouling properties. However, compared with solvent-based coatings, water-based coatings have unique rheological properties, are not volatile, and are more susceptible to environmental temperature and humidity [17]. Therefore, it is particularly important to improve the application efficiency by accelerating the curing speed of the coating.

A microwave is an electromagnetic wave whose frequency is from 300 MHz to 300 GHz. Microwave heating has the characteristics of high efficiency and green [18]. Since Gedye et al. [19] reported that microwave radiation can accelerate the chemical reaction, microwave-assisted organic synthesis has become one of research highlights and has been applied in many fields, such as organic synthesis [19,20], inorganic synthesis [21,22], polymer polymerization [23,24], curing materials [25,26,27]. In terms of cured materials, compared with curing in ambient, the curing cycle of carbon fiber epoxy composites cured by the microwave can be shortened by 83% and will not cause new chemical reactions. Compared with thermal curing, microwave-cured carbon fiber epoxy composites have a higher glass transition temperature and significantly improved interface bonding properties [26]. For poly (ethylene oxide) and silica composite coatings, the microwave curing speed is faster than the thermal curing speed, and the surface friction coefficient of the microwave-cured coating is larger [27]. For waterborne polyurethane coating, microwave irradiation can improve its curing speed, and the curing time is only 1/7 of that of conventional curing, and the samples cured by microwave radiation have better corrosion resistance [28]. Microwave technology is not only used for heating and drying but is also widely used in microwave reaction synthesis, microwave curing, microwave rapid repair, and other industrial fields. For example, LUO et al. [29] studied a new technology for repairing metal structure damage by microwave. For metal structures with damage, the strength is improved after microwave repair of composite materials, and the recovery rate is more than 80%. Ma Shining et al. [30] developed microwave repair devices and designed and developed various forms of microwave radiators. It has been successfully applied to the rapid repair of 45 steel, LY12 aluminum alloy, and epoxy/glass fiber composite with a composite patch. After repair, the static strength recovery rate of the three types of materials was more than 88.7%, 89.4%, and 95.3%, respectively. For 45 steel and LY12 aluminum alloy materials, microwave power of 300 W and curing time of 20 min are the best, and the static strength recovery rates are 96.1% and 102.9%, respectively. For epoxy/glass fiber composites, microwave power of 200 W and curing time of 4 min are the best, and the static strength recovery rate can reach 142.8%.

So far, we have not found relevant papers on microwave curing waterborne silicone coatings; that is, the research in this field is not deep enough. In this paper, a waterborne silicone coating was prepared and cured by microwave. On the one hand, it is expected that microwave irradiation can accelerate the curing speed of the coating to improve the coating efficiency. On the other hand, it is expected that microwave irradiation can change the surface morphology of the coating to improve the performance of the coating.

## 2. Experiment

### 2.1. Materials

DY-OH5011 silicone oil emulsion was purchased from Shandong Dayi Chemical Co., Ltd., Yantai, China. It is used as film-forming material of waterborne silicone coatings. DY-OH5011 is a macromolecule silicone material made from high purity midbody (Octamethyl cyclotetrasiloxane, D4) by emulsion polymerization and its solid content is 30 ± 2 mass %. Silane coupling agent KH570 (γ-Methacryloyloxypropyl trimethoxysilane) was used as a curing agent, which is obtained from Nanjing Jingtianwei Chemical Co., Ltd., Nanjing, China. Dibutyltin dilaurate (DBTDL, AR) was obtained from Tianjin Kemio Chemical Reagent Company and used as a catalyst. *Navicular Tenera* was purchased from the Qingdao Algae Germplasm Resource Bank of the Chinese Academy of Sciences.

### 2.2. Preparation of the Waterborne Silicone Coating

KH570 was added to DY-OH5011, and then the mixture was stirred at 1000 rpm for 5 min in the BGD750 sand grinding dispersion mixing multi-purpose machine (Guangzhou Biuged Laboratory Instrument Co., Ltd., Guangzhou, China). DBTDL was added to the mixture, and it was stirred at 1000 rpm for 5 min to prepare an aqueous silicone coating. In total, 3 mL paint was applied at 76.2 mm × 25.4 mm × 1 mm slide, the sample was immediately placed in the G80F23CN1P-G5(S0) microwave oven (Guangdong Galanz Microwave Oven Electric Appliance Manufacturing Co., Ltd., Guangdong, China, wave frequency 2450 MHz). The samples were cured with 80 W power and microwave time of 1 min, 2 min, 3 min, 4 min, 5 min, and the prepared coatings were named MW1, MW2, MW3, MW4, and MW5, respectively. The sample cured in ambient was used as the controlling sample, and the coating was named RT. All coatings shall be soaked and cleaned in deionized water for 1 h, and subsequent tests shall be carried out after drying.

### 2.3. Testing and Characterization

#### 2.3.1. Infrared Spectrum Analysis

Fourier Transform Infrared Spectroscopy (FTIR) (PerkinElmer Co., Ltd., Waltham, MA, USA) was selected to analyze the chemical structure of the coating. The analytical method is attenuated total reflection (ATR) method. The coating surface was scanned 32 times with a spectral resolution of 2 cm^−1^, ranging from 4000 to 650 cm^−1^.

#### 2.3.2. Surface Morphology Analysis

The surface of the coating was observed by laser confocal scanning microscope OLS4000 (Olympus (China) Co., Ltd., Beijing, China) and the image of the surface morphology of the coating was analyzed by LEXT software (version 2.2.4) to obtain the surface linear roughness (Ra) of the coating. Each sample shall be subjected to 6 roughness measurements and their average value shall be taken as the result. The 3D topology of the measured coating was reported.

#### 2.3.3. Contact Angle Measurement

The droplet angle measurement method was used to measure the water and diiodomethane contact angle on the coating with the instrument JC2000C (Shanghai Zhongchen Digital Technology Equipment Co., Ltd., Shanghai, China). For each sample, 3 points were measured, and the droplet size was 3 μL. The image obtained after the droplet was on the surface for 3 s was measured. Based on two liquid methods proposed by Owens [31], the surface free energy of each coating was calculated with Formulas (1)–(3). In the formulas, $\theta_{H_{2}O}$ is water contact angle; $\theta_{{\mathrm{CH}}_{2}I_{2}}$ is diiodomethane contact angle; $\sigma_{S}^{p}$ is the polar force; $\sigma_{S}^{d}$ is the dispersion force; $\sigma_{S}$ is the surface free energy.

(1) $$\sigma_{s}^{p}=[(137.5+256.1{\;\times \;\cos\theta }_{H_{2}O}\;-\;118.6{\;\times \cos\theta }_{{\mathrm{CH}}_{2}I_{2}})/44.92{]}^{2}$$

(2) $$\sigma_{s}^{d}=[(139.9+181.4{\;\times \;\cos\theta }_{{\mathrm{CH}}_{2}I_{2}}\;-\;41.5{\;\times \cos\theta }_{H_{2}O})/44.92{]}^{2}$$

(3) $$\sigma_{s}{\;=\;\sigma }_{s}^{p}{\;+\;\sigma }_{s}^{d}$$

#### 2.3.4. Tensile Test

Tensile test of the samples was carried out at a tensile rate of 50 mm/min with the electronic universal testing machine UTM5105 (Jinan Wance Electrical Equipment Co., Ltd., Jinan, China). The coating on the slide was taken off and made into the 75 mm × 25 mm × 4 mm dumbbell-shaped specimen according to ISO 37:2005 [32]. The tensile data whose strain did not exceed 0.02 mm/mm was selected for linear fitting, and the slope fitted was taken as the elastic modulus. Each coating was subjected to 6 tensile tests and their average value was taken as the result.

#### 2.3.5. Marine Bacterial Attachment Test

The marine bacterial attachment test was used to evaluate the anti-marine bacterial attachment properties of the coating. Six samples for each coating cured were immersed in natural seawater for 24 h. The seawater was taken from the Yellow Sea area of Dalian. After immersion, three samples were rinsed in 50 mL of sterilized seawater. The rest threes were put into a centrifugal tube containing 50 mL sterilized seawater for oscillation. The oscillation time was 20 min, and the frequency was 130 r/min to simulate the washing of seawater flow on the coating. Then, the bacteria on the coating surface were brushed into sterilized seawater with a cotton swab to make a bacterial suspension. The suspension was diluted to 10^6^ times by sterilized seawater, and 10 μL of suspension was inoculated on the solid medium 2216E. The solid medium was placed in SHP-080 biochemical incubator (Shanghai Jinghong Experimental Equipment Co., Ltd., Shanghai, China) for 48 h, and the colony data were photographed and recorded. Then, the bacterial colony area on the 2216E was counted by Image Pro Plus (version 6.0) software. The antifouling performance of waterborne silicone coatings was evaluated according to the proportion of bacterial community area in the entire solid medium area. The relative change in colony area after rinsing and washing of the same sample represented the removal rate of bacteria [29].

#### 2.3.6. *Navicular Tenera* Attachment Test

Six samples were selected from each coating and placed in *Navicular Tenera* suspension. Then, they were placed in the HPG-280BX (Harbin Donglian Electronic Technology Co., Ltd., Harbin, China) light incubator for 48 h. The temperature of the light incubator was set to (18 ± 1) °C and the light-dark ratio was 12 h:12 h. Then the samples were taken out, half of the samples were rinsed, and the other half washed. The washed and rinsed method is same as reported in Section 2.3.5. The evaluation method of *Navicular Tenera* attachment is detailed in the paper [33].

## 3. Results

### 3.1. Microwave Curing Effect and Mechanism

**Figure 1:**
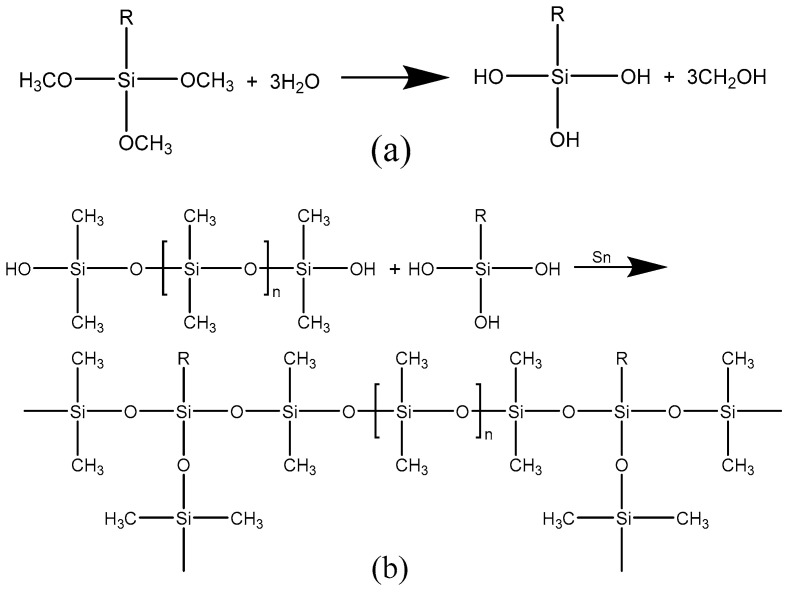
Chemical reaction principle diagram of waterborne silicone. (**a**) The hydrolysis reaction of curing agent, (**b**) crosslinking reaction between silanol and silicone.

When the coating was cured in ambient at 20 °C, its film-forming time is 30 min. We find that coatings have different curing results with 80 W power and different microwave times. After microwave irradiation for 1 min, the coating is only partially cured on the surface, and the coating can be completely cured after continuous curing in ambient. After microwave irradiation for 2 min, the film-forming area of the coating is significantly improved, but it still needs to be cured in ambient to form a complete film. When the microwave was applied for 3 min, the coating was completely cured. When the microwave is applied for 4 min and 5 min, the coating is completely cured, and there is no significant change in macro compared with 3 min. According to the above results, microwave-assisted curing can greatly improve the film-forming speed of the coating, and the maximum film-forming time of the coating can be reduced to 1/10 of the one cured in ambient. To explore the mechanism of microwave accelerated curing, it is necessary to analyze the chemical reaction principle of waterborne silicone coating, and its chemical reaction principle is shown in Figure 1 [16].

For the coating cured in ambient, KH570 is hydrolyzed in the aqueous system to produce silanol, as shown in Figure 1a. As shown in Figure 1b, a three-dimensional network structure is formed by the crosslinking of silanol and hydroxyl-terminated by silicone due to the action of the catalyst. The R group in the structure is a methacryloyloxy group, which does not participate in the crosslinking reaction [16]. For microwave-assisted curing coatings, the microwave mainly acts on the hydrolysis process. Additionally, the microwave frequency emitted by the microwave oven is 2450 MHz. At this frequency, the water molecules in the coating are rapidly changed in polarity and move at high speed. Subsequently, adjacent water molecules rub against each other to form intramolecular heating, which further accelerates the film formation of the coating. Under the action of a microwave, the reaction activation energy is reduced, and the hydrolysis reaction is accelerated [33,34,35], which accelerates the formation rate of silanol. The subsequent crosslinking reaction is completed in a shorter time, which speeds up the film-forming speed of the coating.

### 3.2. Chemical Structure

**Figure 2:**
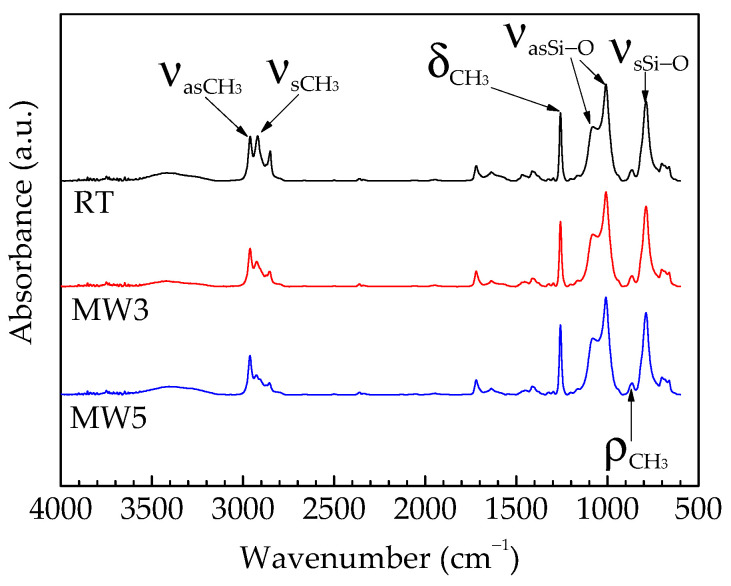
The infrared spectra of the coatings.

The infrared spectrum of the coatings was shown in Figure 2 under different curing conditions. For the cured in the ambient coating, the characteristic peaks that are reflected by the Si-O-Si bond of the main chain of the compound are the antisymmetric stretching vibration peaks at 1085 cm^−1^ and 1020 cm^−1^, and the bending vibration peak at 1258 cm^−1^ is provided by the C-H bond of Si-CH_3_. All the above are the characteristic peaks of silicone. For the coating cured by microwave for 3 min and 5 min, the characteristic peaks of the above silicone do not change; that is, they are silicone coatings, which indicates that microwave irradiation does not change the reaction products.

**Figure 3:**
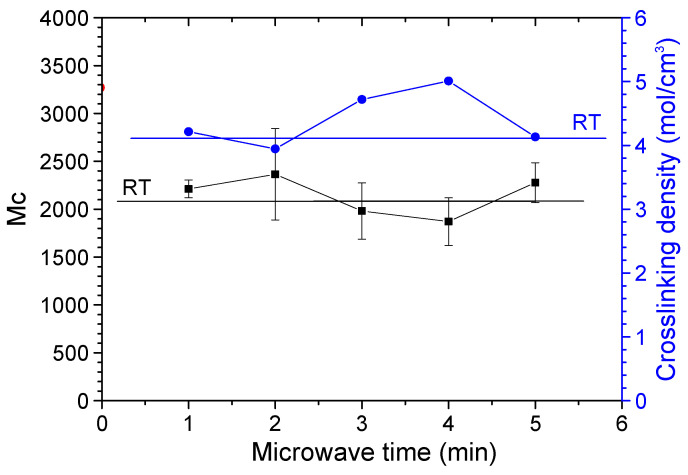
The crosslinking density and Mc of the coatings.

The swelling equilibrium method was used to measure the crosslinking density of the coating and the relative molecular weight (Mc) between adjacent crosslinking points. The crosslinking density and Mc of the coatings are shown in Figure 3. The results showed that there was no obvious relationship between Mc and the crosslinking density and microwave curing time. They fluctuate within the measurement error range and are close to Mc, the crosslinking density of coatings cured in ambient.

### 3.3. Surface Morphology and Roughness

**Figure 4:**
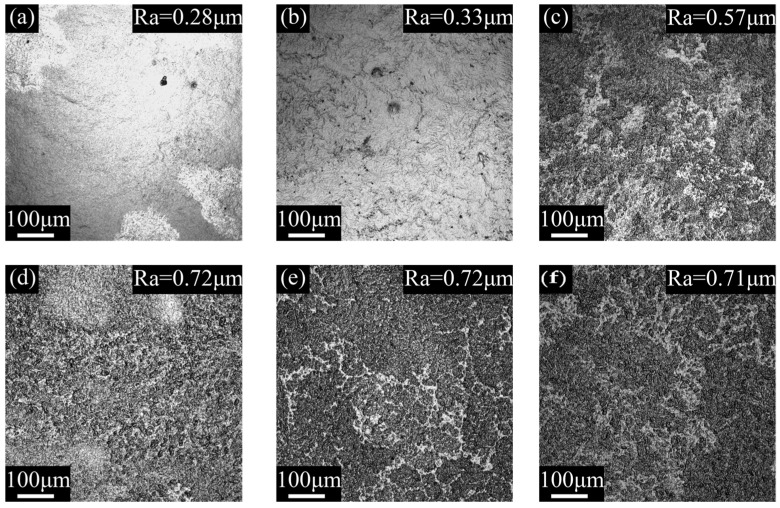
CLSM morphology and roughness of the coating cured at different condition. (**a**) RT (**b**) MW1 (**c**) MW2 (**d**) MW3 (**e**) MW4 (**f**) MW5.

**Figure 5:**
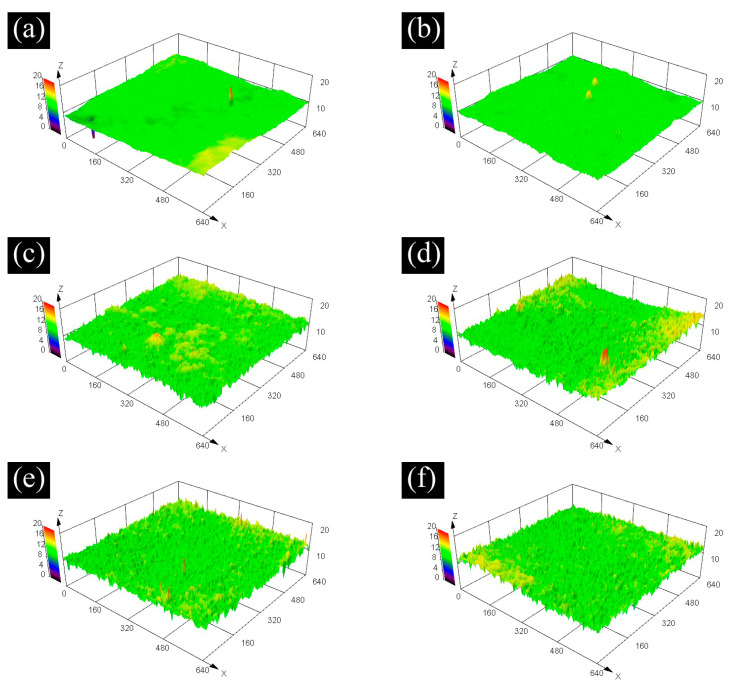
The 3D topography of the coatings cured at different condition (**a**) RT, (**b**) MW1, (**c**) MW2, (**d**) MW3, (**e**) MW4, and (**f**) MW5.

The surface morphology and roughness of the coating under different curing conditions are shown in Figure 4. The corresponding 3D height diagram of the coating is shown in Figure 5.

According to Figure 4 and Figure 5, for the surface of the coatings cured in ambient, there are several hole defects, and the surface morphology is irregularly arranged. The microstructure of the coating surface is relatively flat, but the overall height fluctuation of the coating surface is large. The surface of a microwave-cured coating tends to be flat, and the hole defects on the surface are significantly reduced, but highly similar “convex structures” begin to appear at the micro size of the coating. The number of such “convex structures” gradually increases with the increase in microwave curing time and reaches its maximum when the microwave curing time is 3 min, and the number will not change significantly. The surface roughness of the coating is directly proportional to the curing time, and the roughness will be stable after the microwave time is more than 3 min. This result is also consistent with the change in morphology of the coating surface.

The change in the surface morphology of the coating is affected by the action of the microwave. The hydrolysis speed of the coating cured in ambient is slow, which leads to a relatively slow generation speed of silanol. Simultaneously, there is no external heating. Therefore, the coating is slowly and gradually cured, which makes the curing of the coating uneven, generates stress inside the coating, and causes height drops and defects on the coating surface. It can be seen from Section 3.1 that compared with the coating cured in ambient, microwave promotes the film-forming speed of the coating, which makes the cross-linking curing reaction more uniform and rapid and reduces the internal stress in the curing process of the coating. Therefore, the coating surface after microwave curing is flatter and has fewer defects. The change of “convex structures” and roughness of the coating surface is mainly because of microwave on water molecules. Under microwave irradiation, only the surface part of the coating forms a film at the beginning, and there is the liquid-phase paint under the film. The microwave causes the water molecules in the paint to vibrate violently, which constantly impacts the coating surface. Finally, these micro “convex structures” appear, which improve the roughness of the coating. Additionally, there is a micro-explosion mechanism in the process of microwave curing coating. Because microwave irradiation is a heating method from inside to outside, the internal solvent also volatilizes violently and explodes at the microstructure level. The resin splashes around, leaving a dot shape around and a crater shape on the surface of the film, making the convex structure more obvious, and finally leading to the formation of a rough surface of the coating.

For the coating cured by microwave for 1 min, it only forms a film on the surface layer after microwave action, so there are fewer “convex structures” on the surface, and the change in roughness is not obvious. For the coating cured by microwave for 2 min, the coating after microwave only partially forms a film, so the coating only partially appears as “convex structures”. After the microwave curing for 3 min, the coating has completely formed a film, so the coating surface presents this structure, and the roughness is greatly improved. When the microwave curing time is more than 4 min and 5 min, the roughness change is not obvious. The reason for this phenomenon is that when the microwave curing time is 3 min, the coating has formed a film, so the subsequent microwave action cannot affect the surface morphology of the coating.

### 3.4. Contact Angle and Surface Energy

**Figure 6:**
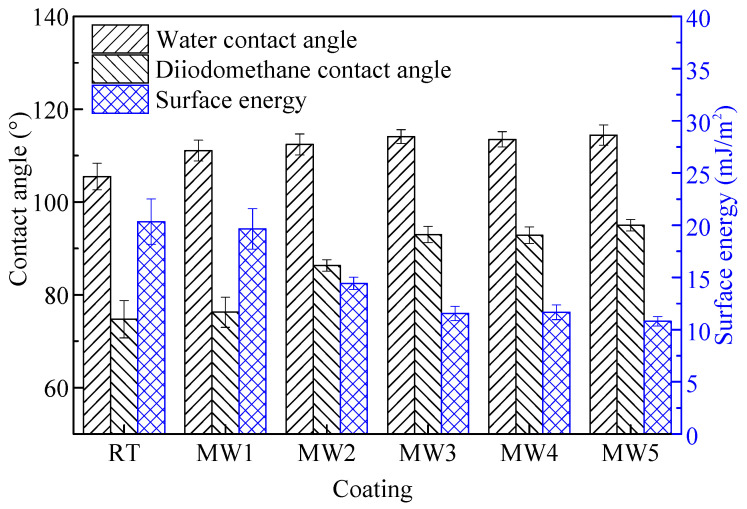
The contact angle and surface energy of the coatings cured at different condition.

The results of the contact angle and surface energy of the coatings are shown in Figure 6. For the coating cured in the ambient, the water contact angle is 105°, which belongs to a hydrophobic coating. The surface energy of the coating is about 20 mJ/m^2^. The hydrophobicity and low surface energy are determined by the characteristics of the film-forming material in the coating. For silicone polymers, the methyl group is linked with the silicon atom on the Si-O-Si main chain by σ bond, and the whole linear molecular chain presents a spiral arrangement, isolating the Si-O-Si bond from the outside. Because the methyl group in contact with the outside has strong non-polarity, the coating shows strong hydrophobicity [36].

For the coating cured by microwave, with the increase in microwave curing time, the water and diiodomethane contact angles of the coating gradually increase, and the surface energy gradually decreases. This change lasts until the microwave time is 3 min. After that, the contact angle and surface energy have no obvious change when the microwave time continues to increase. The reasons for this phenomenon are as follows: According to infrared analysis, microwave curing does not change the composition of the coating, so the coating after the microwave is still hydrophobic. According to the analysis of surface morphology, with the increase in microwave curing time, the coating surface gradually forms a micro “convex structure”, which leads to a gradual increase in roughness. For hydrophobic coatings, the higher the roughness is, the larger the contact angle is, which makes the wettability of droplets on the coating surface worse. Therefore, the coating shows a lower apparent surface energy [37]. However, the surface roughness of the coating after microwave cured for 3 min, 4 min, and 5 min does not significantly change, so the contact angle and surface energy have not changed.

### 3.5. Tensile Properties

**Figure 7:**
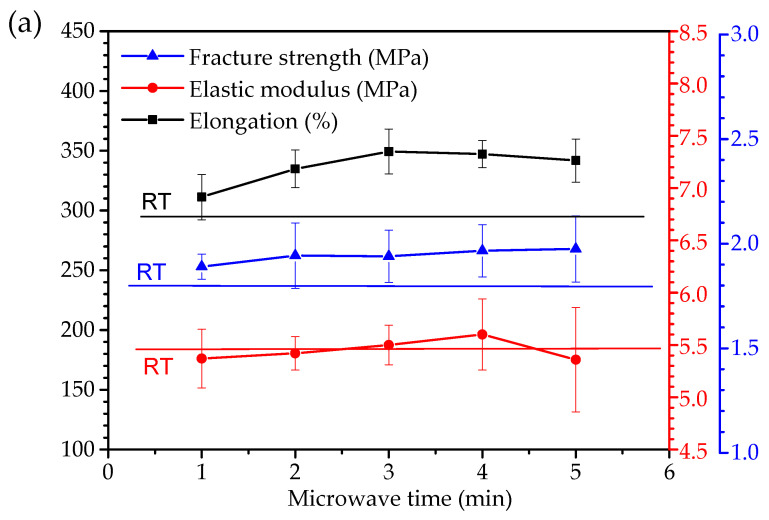

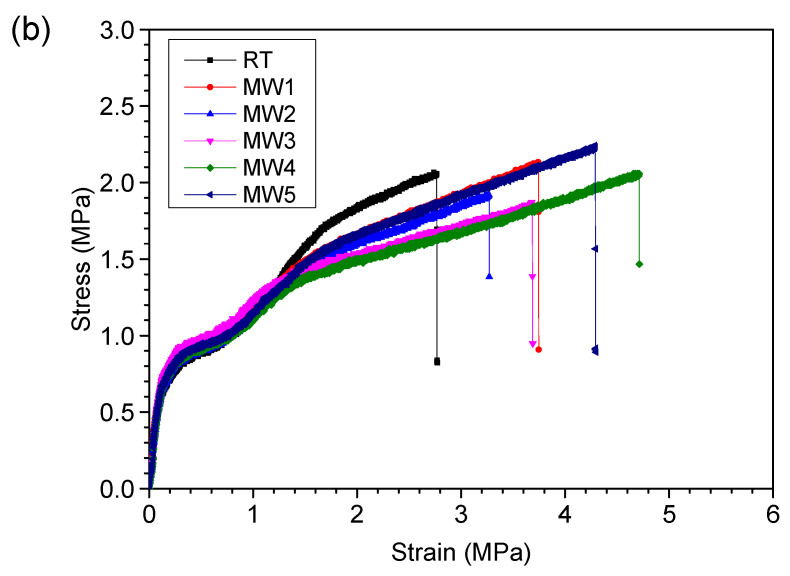
Tensile properties of coatings with different microwave curing time. (**a**) Elastic modulus, breaking strength, and elongation, (**b**) Stress–strain curves.

The relationship between microwave curing time and the tensile properties of the coating is shown in Figure 7a. The stress–strain curves of the coatings are shown in Figure 7b. The horizontal line in Figure 7a represents the property of the coating cured in ambient. The results showed that microwave curing has almost no obvious effect on the elastic modulus of the coating, as shown in Figure 7a. This result is because the microwave only changes the surface morphology of the coating but does not change the reaction products and chemical structure of the coating system, so the elastic modulus of the coating after the microwave is not affected. For the breaking tensile strength and elongation of the coating, they are higher than those cured in ambient. The strength increases slightly with the increase in microwave time. The elongation increases with the increase of microwave time from 1 min to 3 min. The highest reaches 349.3%, then it decreases slightly with the increase in microwave time. This phenomenon is attributed to the change in the surface morphology of the coating by microwave. The improvement of coating performance is attributed to the reduction of coating surface defects by microwave curing, and the gradual disappearance of coating surface defects makes the performance of the coating tend to be stable with the extension of microwave time.

### 3.6. Antifouling Performance

**Figure 8:**
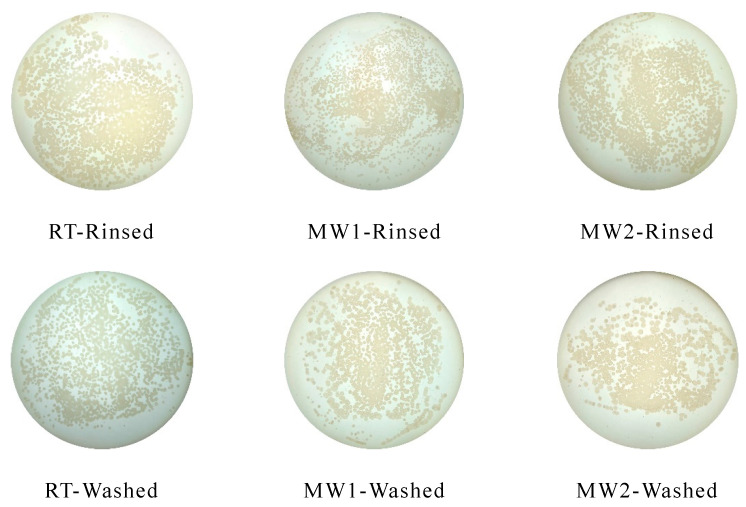

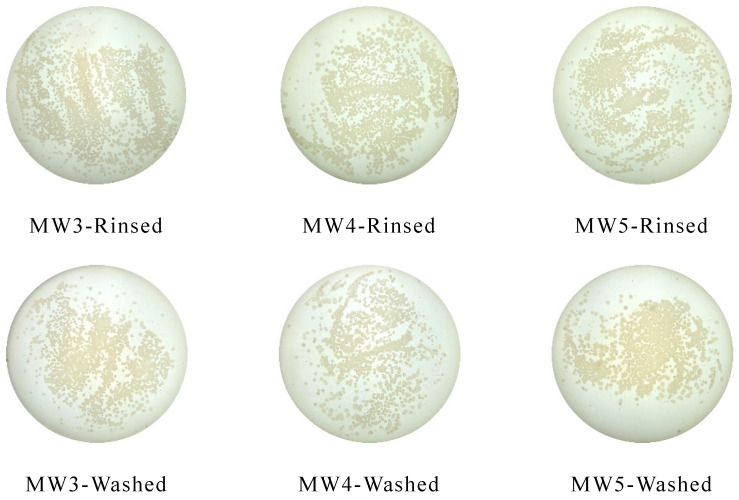
Bacterial colonies images of the cured coating under different conditions.

**Figure 9:**
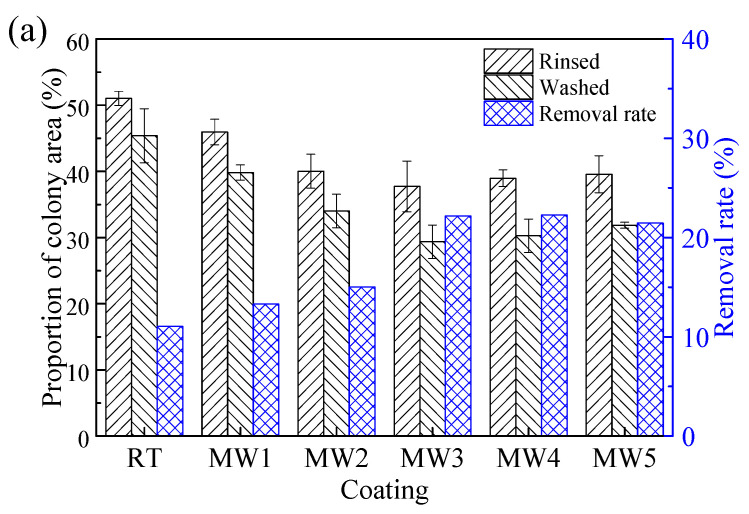

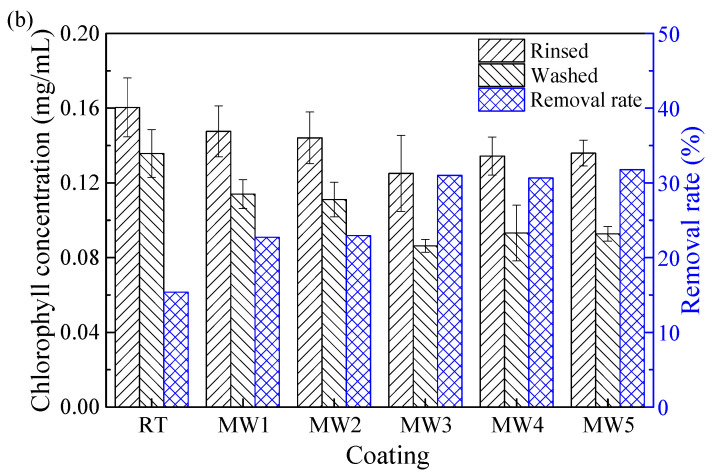
Antifouling performance of the coatings cured at different condition. (**a**) Marine bacteria (**b**) *Navicular Tenera*.

The original colony images of the bacterial adhesion tests were shown in Figure 8, and the quantified colony adhesion and removal rates were shown in Figure 9a.

In Figure 9, under the same rinsing or washing conditions, the bacterial colonies attachment area of the microwave curing coating is significantly smaller than that of the coating cured at ambient. When the microwave time was increased from 1 min to 3 min, the number of bacteria on the coating decreased gradually, and there was no significant change after 3 min. It is affected by the surface energy of the coating. Bacteria are difficult to adhere to or are unstable on low surface energy coatings [38]. So, as the change of the surface energy tends to be stable with the increase in microwave time, as shown in Figure 6, the anti-bacterial adhesion performance of the coating also tends to be stable.

In Figure 9, compared with cured in ambient, the colony area of the washed coating is lower than that of the rinsed coating. This change is quantified as the bacterial removal rate of the coating. As shown in Figure 9a, under the action of the microwave for a certain time, the bacterial removal rate of the coating gradually increases and tends to be stable, which is related to the hydrophobicity of the coating. By comparing Figure 6, the change in coating hydrophobicity and bacterial removal rate is consistent with the change in microwave time. Relevant research showed that for low surface energy coatings, the more hydrophobic the coating is, the better the self-cleaning performance is; that is, the better the stain resistance of the coating is [39]. Specifically, after microwave curing, the bacterial removal rate of the coating can be increased from 11.05% to 22.28%.

The results of the *Navicular Tenera* attachment tests are shown in Figure 9b. Compared with the coating cured in ambient, the coating after microwave curing has better performance. With the increase in microwave time, the attachment amount of *Navicular Tenera* gradually decreases, and the removal rate gradually increases, and the removal rate of *Navicular Tenera* in the coating increased from 15.36% to 31.78%. When the microwave curing time reaches 3 min, it tends to be stable. It can be attributed to the change in hydrophobicity and surface energy of the coating by microwave curing, which makes the coating more hydrophobic and has lower surface energy. This makes biofouling organisms difficult to adhere to on the coating, and the adhesion is easy to remove [40].

## 4. Discussion

### 4.1. Relationship between Surface Energy and Roughness

**Figure 10:**
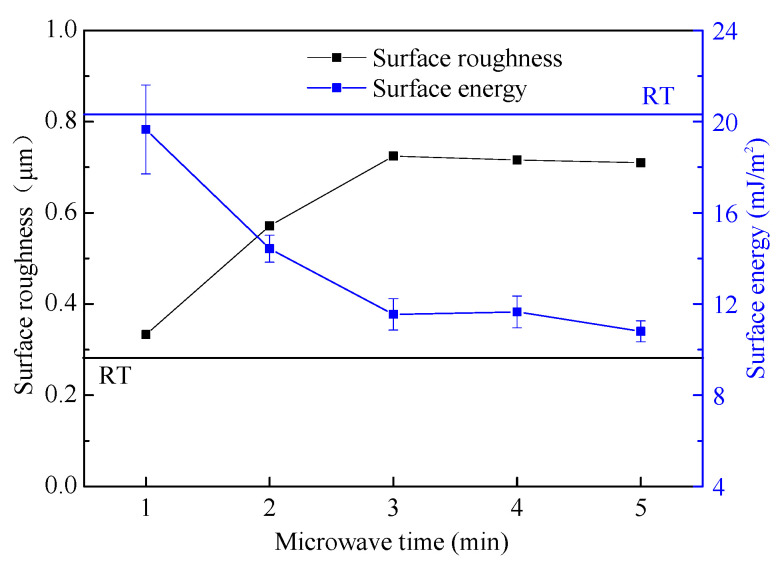
Surface roughness and surface energy of the coatings.

The relationship between the surface energy and the surface roughness of the coating under different microwave times is shown in Figure 10. The horizontal lines in the figure represent the roughness and surface energy of the coating cured in ambient, respectively. The results show that the microwave time is directly proportional to the surface roughness and inversely proportional to the surface energy when the microwave time is 1–3 min. After that, with the extension of microwave time, they have no obvious change.

**Figure 11:**
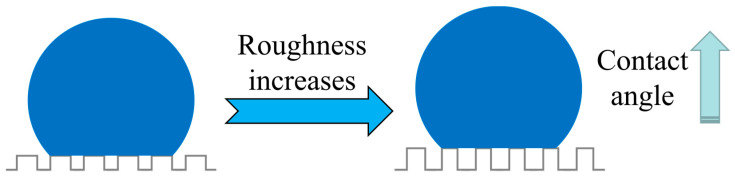
Schematic diagram of contact angle change caused by roughness change.

According to Cassie’s model, when the droplet contacts the coating surface, the air will be trapped in the gap of the surface, and the apparent contact angle of the coating would increase if the air trapped in the gap increases [41], as shown in Figure 11. According to the calculation formula of the Owens two liquid method [31], the larger the apparent contact angle is, the harder the coating wets, and the smaller the apparent surface energy is. For the coating prepared in this paper, as shown in Figure 4, with the increase in microwave time, the micro "convex structure" gradually appears on the surface, and the surface roughness also increases. When the microwave time is 3 min, the change in surface roughness tends to be stable. This change makes the droplets trap more air, which improves the apparent contact angle and reduces the apparent surface energy. When the increase in microwave time cannot further change the surface morphology of the coating, the two properties are also gradually stable. That is, the experimental results in this paper are consistent with Cassie’s model.

### 4.2. Effect of Relative Adhesion Factor (RAF)

**Figure 12:**
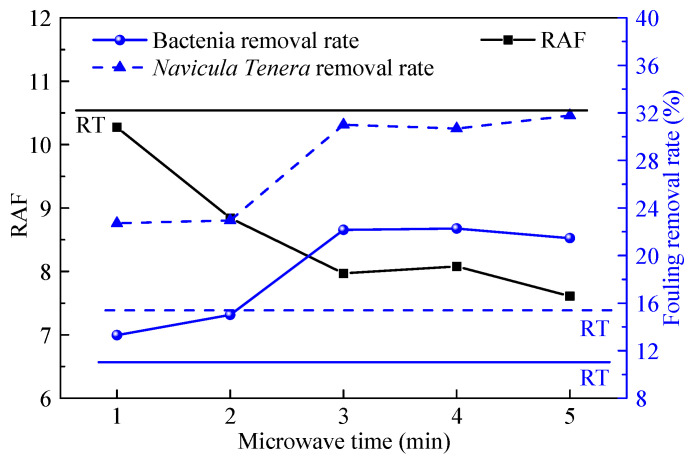
Fouling removal rate and RAF of the coatings at different microwave time.

The relative adhesion factor (RAF) proposed by Brady [42] is an important parameter that can characterize the performance of the coating. The calculation formula of RAF is $\mathrm{RAF}=\sqrt{E\cdot \gamma }$, where *E* and γ are the surface energy and elastic modulus of the coating, respectively. The fouling removal rate and the RAF of the coating in this paper were shown in Figure 12, and the lines represent the corresponding properties of the cured in the ambient coating. The fouling removal rate is inversely proportional to the RAF, which is the same as the theory of Brady. Therefore, according to the relationship between RAF and microwave time, the antifouling effect of the coating is improved after microwave curing.

### 4.3. Relationship between Surface Roughness and Fouling Removal Rate

**Figure 13:**
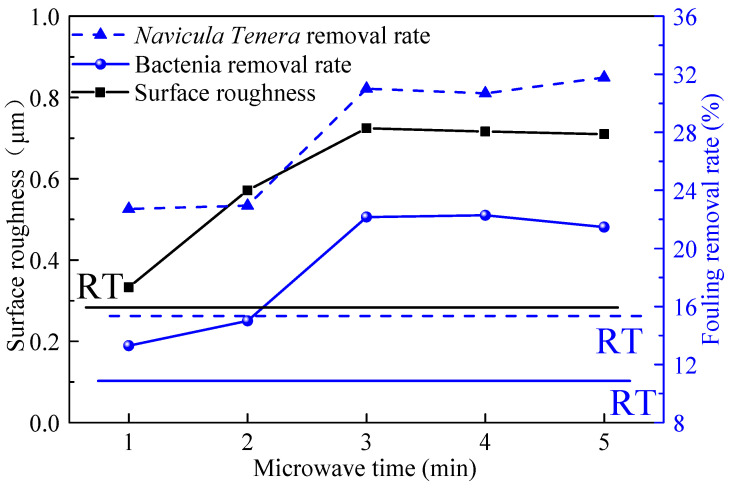
Fouling removal rate and surface roughness of coatings at different microwave time.

The relationship between the surface roughness and the fouling removal rate of the coating under different microwave times is shown in Figure 13. The lines in the figure are the corresponding performance of the cured in the ambient coating. With the increase in microwave time, the changing trend of surface roughness is consistent with the changing trend of the antifouling performance of the coating. According to the above analysis of the Cassie’s model and RAF, microwave curing changes the morphology of the coating and increases the surface roughness, which increases the apparent contact angle of the coating. The change in apparent contact angle reduces the apparent surface energy of the coating, which improves the antifouling performance of the coating. That is, microwave curing can improve the antifouling performance of the coating.

## 5. Conclusions

In this paper, different microwave parameters were selected to cure the waterborne silicone coating, and the effect of the microwave curing time on the properties of the waterborne silicone coating was studied. The results showed that the microwave can greatly reduce the curing time of waterborne silicone coating. Taking 80 W microwave power as an example, the curing time can be reduced from 30 min to 3 min, which can improve the coating efficiency and the surface roughness of the coating. The mechanical properties of the coating have also been slightly improved. The change in roughness increases the contact angle of the coating, reduces the apparent surface energy, and then improves the antifouling performance. For the coating cured by microwave, with the increase in microwave curing time, the water and diiodomethane contact angle of the coating gradually increase, and the surface energy gradually decreases from about 20 mJ/m^2^ to 10.8 mJ/m^2^. With the increase in microwave time, the attachment amount of *Navicular Tenera* gradually decreases and the removal rate gradually increases, and the removal rate of *Navicular Tenera* in the coating increased from 15.36% to 31.78%. The bacterial removal rate of the coating can be increased from 11.05% to 22.28% after microwave curing. Microwave-assisted curing is helpful in improving the antifouling and self-cleaning performance of waterborne silicone coatings, showing promising potential applications.

## Data Availability

The data presented in this study are available on request from the corresponding author.
